# Dr. Bennett’s Operation

**Published:** 1893-06

**Authors:** Will B. Davis

**Affiliations:** City Physician; Pueblo, Col.


### Correspondence.

### DR. BENNETT'S OPERATION.

PUEBLO, COL., April 29, 1893.

*Editor Daniel's Texas Medical Journal:*

To write a criticism on Dr. Bennett's paper, entitled "A Treatment of Fistula in Ano without a Cutting Operation," in your

April edition, would be an unnecessary undertaking in the interest of experienced surgeons, but I wish to say that in my opinion it is not good surgery to go to the extent of divulsing the sphincter, and then substitute an uncertain for a more certain finish. The terror of the knife is all in the imagination of the patient. Thorough divulsion is by far the most severe step in the operation, but neither the divulsion nor the cutting is *severe*, nor should possess any *terrors* under an anesthetic. In short, when you want to cure a fistula in ano, anesthetize patient, divulse the sphincter, cut fistula thorougly through into rectum, remove all pyogenic and callous tissue either by actually dissecting it all out, or scarrifying and curetting thoroughly. Don't do a part of the radical procedure and then switch off on something you should have tried before, if you are going to try it at all. Many recent fistulœ, free of diverticula, may be cured by injecting with carbolic acid or nitrate of silver. If this will not cure without anesthesia and divulsion, when you anesthetize and divulse,—CUT.

WILL B. DAVIS, M. D.,
City Physician.

